# Audience facial expressions detected by automated face analysis software reflect emotions in music

**DOI:** 10.3758/s13428-021-01678-3

**Published:** 2021-09-10

**Authors:** Diana Kayser, Hauke Egermann, Nick E. Barraclough

**Affiliations:** 1 Department of Music, York Music Psychology Group, University of York, Heslington, York, YO10 5DD UK; 2 Department of Psychology, University of York, Heslington, York, YO10 5DD UK

**Keywords:** Emotion, Music, Audience, Facial expressions, Automated facial expression analysis, Basic emotions, Affective responses

## Abstract

An abundance of studies on emotional experiences in response to music have been published over the past decades, however, most have been carried out in controlled laboratory settings and rely on subjective reports. Facial expressions have been occasionally assessed but measured using intrusive methods such as facial electromyography (fEMG). The present study investigated emotional experiences of fifty participants in a live concert. Our aims were to explore whether automated face analysis could detect facial expressions of emotion in a group of people in an ecologically valid listening context, to determine whether emotions expressed by the music predicted specific facial expressions and examine whether facial expressions of emotion could be used to predict subjective ratings of pleasantness and activation. During the concert, participants were filmed and facial expressions were subsequently analyzed with automated face analysis software. Self-report on participants’ subjective experience of pleasantness and activation were collected after the concert for all pieces (two happy, two sad). Our results show that the pieces that expressed sadness resulted in more facial expressions of sadness (compared to happiness), whereas the pieces that expressed happiness resulted in more facial expressions of happiness (compared to sadness). Differences for other facial expression categories (anger, fear, surprise, disgust, and neutral) were not found. Independent of the musical piece or emotion expressed in the music facial expressions of happiness predicted ratings of subjectively felt pleasantness, whilst facial expressions of sadness and disgust predicted low and high ratings of subjectively felt activation, respectively. Together, our results show that non-invasive measurements of audience facial expressions in a naturalistic concert setting are indicative of emotions expressed by the music, and the subjective experiences of the audience members themselves.

Emotional experiences are one of the main reasons for people to engage in music listening (Lamont & Webb, 2010). However, to date, the majority of music and emotion research has been conducted in laboratory settings. Although this allows researchers to study emotional responses to music in a controlled environment, the variety of contexts in which music is experienced is much more diverse. Further, listening experiments are usually completed individually, which makes results poorly generalizable to experiences in everyday life, where many listening experiences take place with other people present (North, 2004), for example at a live concert. Sharing such an experience with other audience members as well as attending a music performance in an ecologically valid setting, such as a concert hall, might not only influence, but even intensify emotional experiences in listeners. Gabrielsson (2010, 2011) was one of the first to provide tangible evidence for this phenomenon. Through qualitative analysis of personal accounts of close to 1000 respondents, he identified that strong experiences with music occur more frequently when listening with others than when listening alone. Many respondents specifically reported having had these experiences in concert halls. These findings were replicated by Lamont (2011), who further argued that the shared environment contributed to the intensity of the overall experience. This explanation is supported by Garrido and Macritchie (2018) who surveyed audience members at three concerts and found that audience member emotional experiences were intensified by the presence of other attendees. The authors further showed that social bonding (i.e., the connectedness audience members felt with others) influenced the emotional experience in an audience. In contrast, however, in a controlled laboratory experiment, Egermann et al. (2011) found no evidence that, compared to a solitary listening condition, emotional experience intensified when participants were experiencing music together as a group. Here, the group condition was somewhat artificial and different to a concert experience; participants knew each other (they were members of the same ensemble), were seated in a circle and listened to music from loudspeakers while also having sensors attached to their fingers, which does not resemble a natural group listening situation. These findings suggest that the presence of others is not the only factor involved in a shared listening experience, such as a concert. Indeed, this conclusion was also reached by Pitts et al. (2013), who found that individual differences in the form of musical preferences and familiarity with the repertoire also play a role.

Whereas most studies have relied on self-reported experience, research has recently started to look at the behavior of audience members during a live concert. Swarbrick et al. (2019) used optical motion capture to investigate the effect of the presence of a performer on audience engagement and found that audience head movements were faster during a live concert when compared to a pre-recorded concert. Seibert et al. (2019) videoed bodies of both audience and performers, and although they found a small to medium synchronization of body movement within the audience, these observations do not necessarily allow insights into the audience subjective experience. This could potentially be better achieved by observing facial expressions of emotion rather than body movement *per se,* as facial expressions are correlated with subjective emotional experiences (see e.g., McIntosh, 1996 for a comprehensive review). The study we present here, for the first time, uses automated face analysis software to explore emotional experiences in audience members and explores the potential to predict subjective emotion ratings based on distinct facial expressions of emotion.

## Music-induced emotion

The definition of ‘emotion’ is currently debated, and a variety of theoretical emotion models have been proposed. One important theory is the component process model (CPM, Scherer, 2004, 2005), which suggests the involvement of several components in emotional experiences, including: cognitive appraisal, physiological arousal, motor expression, and subjective feeling. According to this model, synchronized changes in these components lead to an emotion experience. The majority of studies in music and emotion have focused on the subjective feeling component (e.g., Eerola & Vuoskoski, 2013; Zentner & Eerola, 2010); however, in some studies, physiological arousal and expressive behavior have also been measured (Eerola & Vuoskoski, 2013; Egermann et al., 2013). Emotions in music have been described as those that are recognized in (or thought to be expressed by) the music, and emotions that are subjectively felt (or induced) as a result when listening to music. Although these two responses can overlap (Egermann & McAdams, 2013; Evans & Schubert, 2008; Gabrielsson, 2002; Kallinen & Ravaja, 2006; Schubert, 2013), their independence has to be acknowledged. Empirical evidence that these two phenomena involve different underlying processes (Evans & Schubert, 2008) comes for example from Dibben (2014) who found that increased levels of arousal resulted in higher intensity ratings for experienced emotions but had no effect on ratings of the emotion thought to be expressed by the music. Distinguishing between these two phenomena is critical, as some research methods may be suitable for measuring one phenomenon but not the other (Kayser, 2017).

In this study, we focus on music-induced emotions in audiences and specifically explore both motor expression and subjective feeling in Scherer’s CPM (Scherer, 2004, 2005) by evaluating whether distinct facial expressions of emotion detected by automated face analysis software reflect the emotions in music and can be used to predict ratings of felt activation and arousal. In order to test this, we follow our earlier suggestion to examine video recordings of facial expressions and determine the emotional state of individuals based on facial expressions detected by automated face analysis software.

## Emotion and facial motor expressions

Specific facial muscular patterns are strongly related to basic emotions (Ekman, 1992; Ekman & Friesen, 1971) and it has been suggested that musical characteristics we perceive as emotionally expressive may lead to spontaneous and automatic motor expressions through emotional contagion (Garrido & Schubert, 2011; Juslin & Västfjäll, 2008). Two facial muscles of particular interest are the zygomaticus major, which is involved in smiling and associated with positive valence, and the corrugator supercilii, which is involved in frowning and associated with negative valence (Cacioppo et al., 2008; Dimberg et al., 2000; Larsen et al., 2003). Activation of these muscles is commonly measured using facial electromyography (fEMG), which uses surface electrodes attached to the skin over specific muscle regions. The magnitude of muscle activity then can be inferred from electrical signals picked up by the electrodes.

Facial EMG has been used to explore the relationship between emotional singing and facial muscle activation. Livingstone et al. (2009) found higher activation in corrugator supercilii when participants sung a sad piece and higher activation in zygomaticus major when they sang a happy piece. Indeed, similar muscle activation patterns also occur when participants observe audio-visual recordings of emotional vocal performances (Chan et al., 2013). The sight of the performer’s movements may also have had an effect on facial motor mimicry, although similar effects of only auditory stimuli have also been observed (Bullack et al., 2018; Lundqvist et al., 2009; Witvliet & Vrana, 2007).

Together, these studies show that music categorized as positive and negative produces facial muscle activation of congruent valence. A closer look at the evidence, however, reveals a number of issues, the most critical being that corrugator supercilii activity, in addition to sadness, has also been reported in studies of fear (e.g., Dimberg et al., 1998), disgust (e.g., Rymarczyk et al., 2019), and anger (e.g., Dimberg & Petterson, 2000; Jäncke, 1996). With one facial muscle being involved in several different emotion expressions, it is difficult to be certain which specific emotion was experienced. The application of automated facial expression analysis might help to overcome this limitation given that it is possible to measure activity in multiple different facial muscles simultaneously. Furthermore, facial EMG inevitably draws the participants’ attention to their face, which may impact the participants’ experience of the performance. Automated facial expression analysis is less intrusive than facial EMG and does not need additional preparation (e.g., attaching electrodes) and thus may overcome demand characteristics inherent in fEMG studies.

## Automated facial expression analysis

In the past decade, algorithms that classify distinct facial expressions of emotion in still images and video recordings have been introduced and integrated in commercially available software solutions. One example is *FaceReader* (Noldus, 2016), which uses an artificial neural network to classify six basic emotion categories (happy, sad, anger, surprise, fear, disgust) and neutral expressions. *FaceReader* has been used in a variety of research disciplines and has recently been validated against the Facial Action Coding System (Skiendziel et al., 2019), a manual scoring method developed by Ekman and Friesen (1978). The Facial Action Coding System (FACS) assesses the movement of 44 muscles (Mauss & Robinson, 2009) and served as the foundation for the development of *FaceReader*’s algorithm.

Although automated face analysis software has been used extensively in other contexts such as marketing (e.g., Barreto, 2017), educational research (e.g., Harley et al., 2012), and psychology (e.g., Chóliz & Fernández-Abascal, 2012), to the best of our knowledge, to date it has been used in only one study of music-induced emotion (Weth et al., 2015). Here, they asked participants to bring a sad piece of music that has a strong emotional impact on them, and which others also would describe as sad. Subsequently they compared participant responses while they listened alone to happy and sad music both self-selected and selected by the experimenters. Compared to the happy and sad music selected by the experimenters, participants displayed more facial expressions of sadness when listening to self-selected sad music, no differences were found in happy facial expressions. Although Weth et al. (2015) carried out their study in a controlled laboratory setting, the artificial context may have had an influence on participants’ emotional responses and behavior. First, the experimenter-selected pieces were both instrumental, whereas 94% of the pieces selected by participants contained lyrics which could have contributed to their experience. Second, the participants listened to music alone, and emotional responses to music in the company of others can differ from experiences one has when listening to music alone.

## Aims and objectives

The overall aim of this experiment was to investigate if automated face analysis software can measure emotional expressions of an audience in an ecologically valid classical concert environment. We had three specific questions we wished to address: 1. Can *FaceReader* detect facial expressions of an audience when individuals are free to move as they wish, and in a setting with relatively poor lighting conditions compared to a lab environment? 2. Does the emotion expressed by the music predict specific audience facial expressions? 3. Can we use information from facial expressions to predict audience reports of music-induced pleasantness and activation?

We organized a solo piano recital in which four musical pieces – two unambiguously expressive of sadness, and two unambiguously expressive of happiness – were performed live. Participants were filmed during the concert, and ratings for subjectively experienced activation and pleasantness were obtained for all four pieces at the end of the concert. Facial expressions were analyzed using automated face analysis software and compared with audience self-reports. Participants rated felt experiences on two dimensions rather than a number of emotion categories as participants had to provide ratings after the concert rather than after each piece. As participants had to rely on their memory, we reasoned that this task would be easiest and thus provide the most accurate estimate of their felt experience. According to Russell (1997), any emotional stimulus can be placed in the two-dimensional emotion space and these dimensions have repeatedly been used to study emotional experiences in response to music (see e.g., Eerola & Vuoskoski, 2013). Further, Russell and Bullock (1986) found that these pleasure-arousal dimensions are used to interpret facial expressions of emotion. So, although the measures of felt experience did not use the same words describing emotions as the *FaceReader* software and the way we have described the music pieces, they refer to the same underlying fundamental dimensions of emotion.

## Methods

### Participants

Participants were recruited via mailing lists, word of mouth, and leaflets. As musical preference influences emotional experiences in response to music (Kreutz et al., 2008), participants were screened with the help of an online questionnaire before taking part to ensure they had a preference for classical music, and further that they were willing to be filmed. We subsequently invited 50 participants to take part in the main experiment, and they received £10 for their participation. A power analysis was not conducted, as no previous research was directly comparable. Given Weth et al. (2015), who tested similar hypotheses, found significant effects in a sample of 18 participants, we concluded that our final sample size, in combination with more robust statistical analyses (linear mixed effects models) would be more than sufficient to measure any predicted effects. All participants were naïve to the purpose of the study. The experiment was approved by the Arts and Humanities Ethics Committee, University of York, and was performed in accordance with the ethical standards laid down in the 1990 Declaration of Helsinki.

### Stimuli

For the experiment, we aimed to select four pieces of music that met the following criteria: 1.) Two pieces clearly expressing happiness, and two pieces clearly expressing sadness; 2.) The duration of each piece should not exceed 6 min and 3.) The pianist had to be able to play the pieces. To ensure that the third criterion was met, the musical material was selected by the pianist herself with the other criteria in mind. The pieces are listed in Table 1 and were performed live on a piano by an experienced internationally recognized pianist.

**Table 1:** Composer / title, emotion conveyed, and duration of pieces performed

Order of presentation	Composer *Piece*	Emotion expressed	Duration (mm:ss)
1	Claude Debussy *Arabesque No. 2. (c.1888-1891)* *Allegretto Scherzando*	Happy	03:34
2	Ludwig van Beethoven *Piano Sonata No. 14, Quasi una fantasia, Op. 27, No. 2 (1801) I. Adagio Sostenuto*	Sad	05:54
3	Frédéric Chopin *Étude Op. 10, No. 9 (1892)*	Sad	04:34
4	Wolfgang Amadeus Mozart *Piano Sonata No. 16 in C major, K. 545 (1788) I. Allegro*	Happy	04:31

To confirm that the pieces selected for the main experiment differentially expressed happiness and sadness as intended, we carried out an online experiment to test the degree to which each music piece expressed seven different emotions (sadness, happiness, anger, fear, surprise, disgust, and tenderness). Fourteen participants completed an online questionnaire that was implemented in Qualtrics (Provo, UT, 2019). The questionnaire was sent out via mailing lists and advertised on social media. As the online experiment and the concert experiment were advertised via different channels, it was unlikely that the same participants took part in both studies, although this was not measured directly. Audio recordings of the four pieces made during the live concert experiment were embedded in the online form. The pieces were presented in a randomized order, and then after each piece, seven sliders were presented and used by participants to indicate the degree to which the music expressed each of the seven different emotions on scales ranging from 0 (not at all) to 100 (extremely). To help validate that the emotions expressed by the music were unambiguous, participants were further asked to indicate how confident they were with each of their ratings (see Figure S1 in the supplemental materials https://osf.io/765km/download for descriptive results). Finally, as we were also interested in their subjective felt experience, participants rated on two scales how activated (– 5 = calm to +5 = excited) and pleasant (– 5 = unpleasant to +5 = pleasant) they felt after each piece. Participant demographic information was not collected.

All statistical analyses reported in this paper were carried out in SPSS. All figures were generated using the results obtained by SPSS or the raw data using the ggplot2 package (Version 3.3.3) in R (Version 3.6.1).

**Fig. 1:**
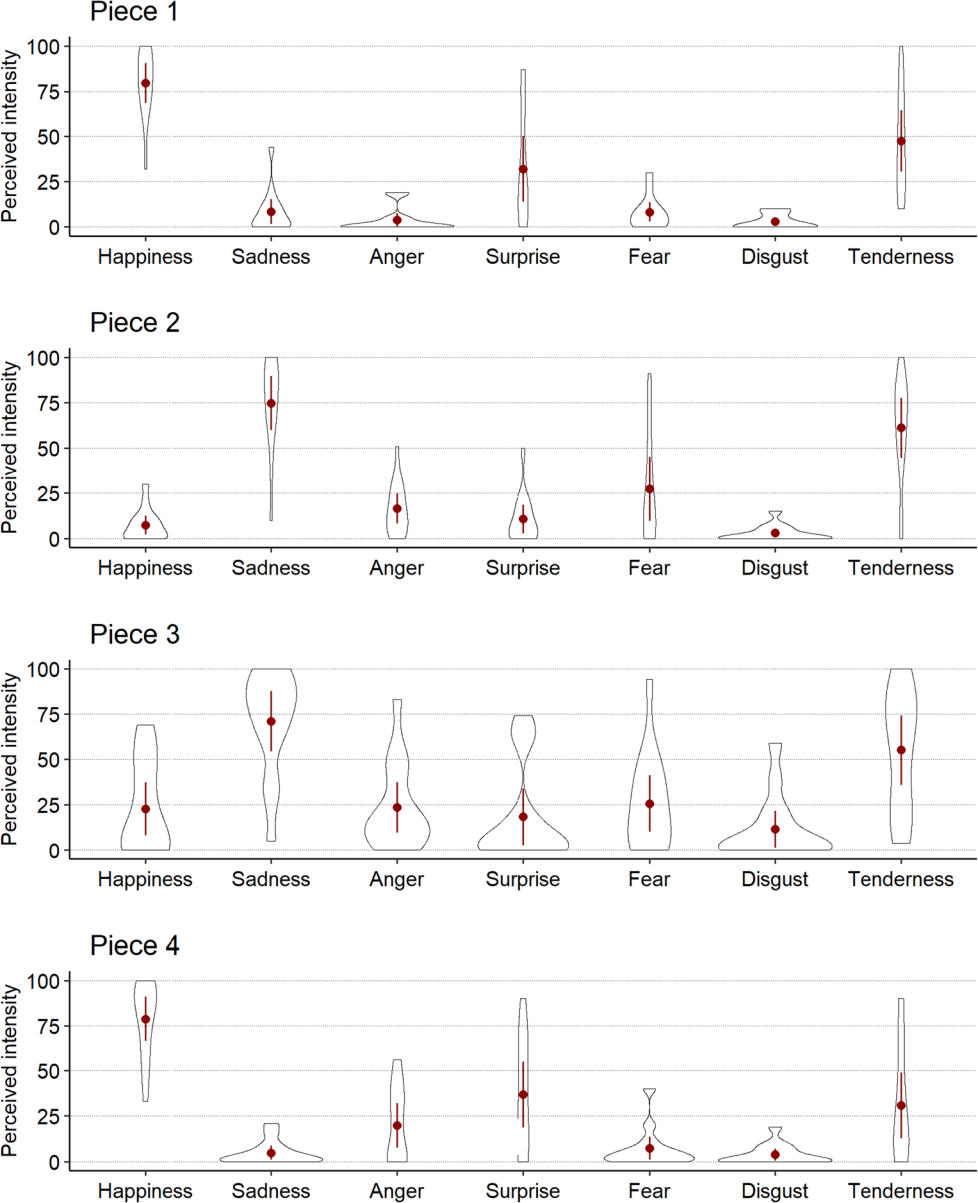
Listeners’ ratings based on self-reports of perceived emotion intensity for each piece. For each violin plot, the outline illustrates data probability density, i.e., the width of the area represents the proportion of the data located there. The *red central dots and whiskers* illustrate the mean perceived intensity and 95 % confidence intervals for each emotion

For each music piece, we conducted separate repeated measures ANOVAs to assess whether participants rated emotions expressed by the music differently. As Mauchly’s tests revealed that the assumption of sphericity had not been met in all four analyses, Greenhouse Geisser corrections were applied. A Bonferroni correction was applied to control for multiple comparisons. Ratings of the emotions expressed (see Fig. 1) were significantly different from each other in each piece (see Table 2).

**Table 2:** Repeated-measures ANOVAs for self-reported expressed emotion for the four pieces

Measure	Effect	*MS*	*df*	*F*	*p*	*η*_*p*_^*2*^
Piece 1	Emotion	30027.34	2.33	34.48	< .001	.726
	Error	870.93	30.31			
Piece 2	Emotion	22104.78	3.02	29.10	< .001	.691
	Error	759.64	39.26			
Piece 3	Emotion	13646.52	2.92	10.14	< .001	.438
	Error	1345.24	37.93			
Piece 4	Emotion	22291.47	2.67	25.20	< .001	.660
	Error	884.45	34.77			

Note: η_p_^2^ = partial Eta squared, Greenhouse–Geisser corrections were applied where appropriate, and the Bonferroni method was used to adjust *p* values for the four tests

To test whether the ‘intended’ emotion expressed (see Table 1) was rated significantly greater than the other emotions, we carried out Sidak-adjusted post hoc tests. Results are reported in Table 3 and show that for all four pieces intensity ratings for the intended emotion were significantly higher than for other emotions, with the exception of ratings for tenderness for pieces that expressed sadness. Ratings of tenderness for music expressive of moderate sadness is not uncommon and has been shown by Eerola and Vuoskoski (2011). However, as both tenderness and sadness were significantly different from happiness, we conclude that the pieces selected for the main experiment differentially expressed happiness and sadness as intended.

**Table 3:** Contrasts between intended emotion and other emotion

Piece	Intended emotion (I)	Other emotion (O)	Mean Difference (I-O)	SE	95% CI Lower	95% CI Upper	*t*	*p*(*t*)
1	Happiness	Sadness	71.1	6.95	49.1	93.2	10.231	< .0001
		Anger	75.8	6.95	53.7	97.9	10.899	< .0001
		Surprise	47.6	6.95	25.5	69.7	6.841	< .0001
		Fear	71.2	6.95	49.1	93.3	10.242	< .0001
		Disgust	76.4	6.95	54.3	98.5	10.992	< .0001
		Tenderness	32.1	6.95	10.1	54.2	4.623	.0004
2	Sadness	Happiness	67.4	7.39	43.95	90.9	9.124	< .0001
		Anger	58.3	7.39	34.81	81.8	7.886	< .0001
		Surprise	64.0	7.39	40.52	87.5	8.660	< .0001
		Fear	47.3	7.39	23.81	70.8	6.398	< .0001
		Disgust	71.8	7.39	48.31	95.3	9.713	< .0001
		Tenderness	13.6	7.39	-9.91	37.0	1.836	.825
3	Sadness	Happiness	48.1	9.67	17.4	78.9	4.980	.0001
		Anger	47.4	9.67	16.6	78.1	4.899	.0001
		Surprise	52.6	9.67	21.9	83.3	5.438	< .0001
		Fear	45.2	9.67	14.5	75.9	4.677	.0003
		Disgust	59.4	9.67	28.6	90.1	6.140	< .0001
		Tenderness	15.9	9.67	-14.9	46.6	1.640	.930
4	Happiness	Sadness	73.9	7.5	50.1	97.8	9.851	< .0001
		Anger	58.9	7.5	35.1	82.8	7.852	< .0001
		Surprise	41.9	7.5	18.0	65.7	5.577	< .0001
		Fear	71.5	7.5	47.7	95.3	9.527	< .0001
		Disgust	75.0	7.5	51.2	98.8	9.994	< .0001
		Tenderness	47.9	7.5	24.0	71.7	6.377	< .0001

Note: Degrees of freedom (*df*) was estimated using Kenward–Roger approximation. For all tests, *df* = 78. Sidak method was used to adjust confidence levels and *p* values for 24 tests.

**Fig. 2:**
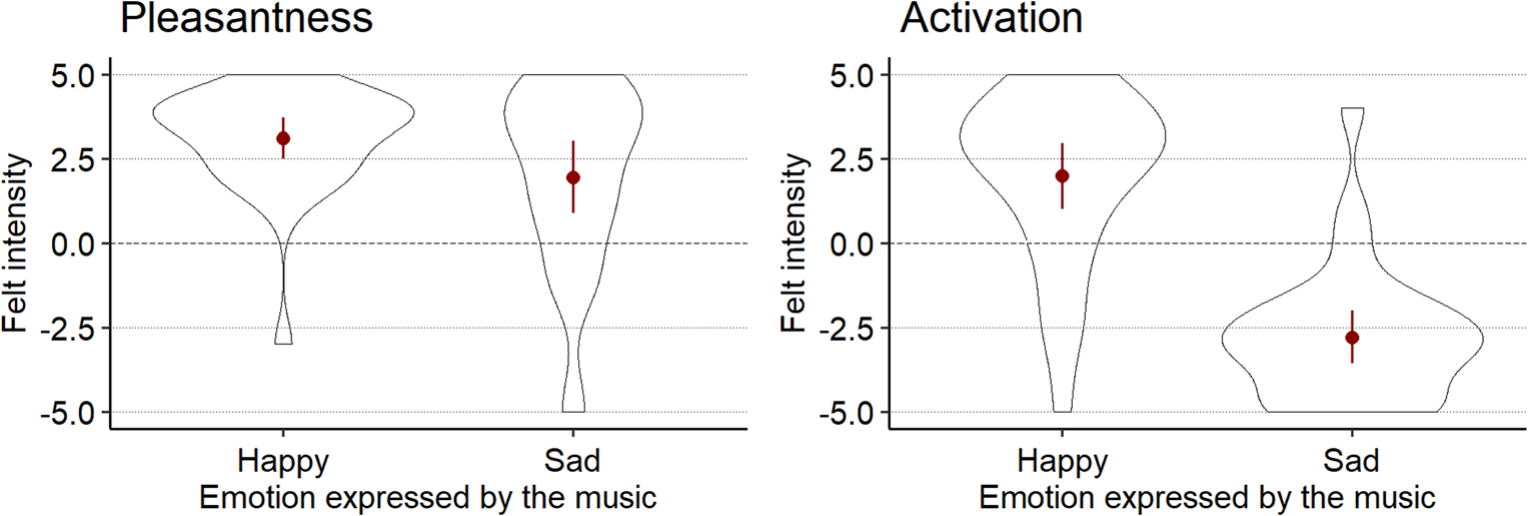
Online listeners’ ratings based on self-reports of felt pleasantness (*left*) and activation (*right*) as a function of emotion expressed in the music. For each violin plot, the outline illustrates data probability density; the *red central dots and whiskers* illustrate the mean felt intensities and 95% confidence intervals

We evaluated whether ratings for pleasantness and activation (see Fig. 2) differed between “happy” and “sad” music. We explored this by fitting separate hierarchical linear models for both dependent variables separately. In each model, participant identity was the upper level (“subjects” in SPSS MIXED) whereas the name of each piece was defined as the lower level (“repeated”). A new grouping variable “emotion expressed” which codes whether a piece expressed happiness or sadness was used as a fixed factor. The covariance structure with the best model fit (as determined by Akaike’s Information Criterion, AIC) was Compound Symmetry Heterogenous (CSH). Type III tests of fixed effects showed that the emotion expressed in the music had a significant main effect on both pleasantness (*F*(1, 32.66) = 5.11, *p* = .03) and activation (*F*(1, 29.84) = 130.85, *p* < .001).

### Procedure

The experiment was carried out in the Arthur Sykes Rymer Auditorium at the Music Research Centre at the University of York. This performance space is frequently used for concerts and events as well as research purposes and is therefore ideal for studying an audience in an ecologically valid environment. The auditorium consists of 138 seats in total.

Follow-up e-mails were sent to participants 1 week as well as 2 days prior to the experiment, asking participants to confirm their attendance to ensure concert attendance. When participants arrived at the venue, they signed a consent form and received a participant information sheet before they were followed into the auditorium by a research assistant. Participants were instructed that they could sit wherever they wanted as long as their seat was within the area filmed by the video cameras. All participants received a concert program which contained the titles of the pieces, names of the respective composers, and the order in which they were performed. Once all participants sat down, they received oral instructions about the procedure from one of the authors, and the concert started immediately afterward. The four pieces were played in succession with a short pause in between them. Participants did not clap after the end of a piece although they did not receive any instructions about clapping or any kind of behavior. After the last piece, however, participants applauded.

In order to record facial expressions participants were filmed with four digital video cameras (3 Panasonic HDC TM900, 1 Panasonic HDC SD90) in full High Definition (1920 x 1080) at 50 frames per second. The cameras were mounted above the performance space, with fields of view in different sections of the seating area. Each camera could view 15 seats (three rows, 5 seats) with an overlap of one row. As automated face analysis software relies on video recordings made under good light conditions (Abbasi et al., 2013), the light was left on in the concert hall during the experiment. The lights were mainly coming from the ceiling as the architecture of the room did not allow us to install additional lights.

As the cameras were installed at a distance of ca. 10–25 m from the participants (depending on where they were seated), we tested the camera setup to see whether the software would pick up facial expressions. To test this, one of the researchers and a research assistant sat in different seats and recorded several facial expressions, which then were analyzed using automated face analysis software (see section Data analysis below). These tests were successful.

To limit participants’ attention to their own faces, we did not ask them to pose for a ‘neutral’ expression baseline video recording before the concert. Furthermore, as participants may experience a range of emotions during the pre-concert period, including excitement, boredom, or interest in the study itself, expressions here may not truly represent a neutral emotional state.

To not interfere with the concert experience, we administered questionnaires after the last piece had finished, while participants were still in their seats. The questionnaire tested their emotional experiences; in addition, questions about their liking of the music as well as their familiarity with the pieces were also administered as part of another study and are not discussed in this paper (descriptive statistics can be found in Table S2 in the supplemental materials https://osf.io/765km/download). Self-reports of experiences of pleasantness and activation during the four music pieces were obtained via a questionnaire presented either on an iPad via Qualtrics (Provo, UT, 2019) or on a piece of paper. For each of the four pieces, participants rated how pleasant (– 5 = unpleasant to +5 = pleasant) and activated (– 5 = calm to +5 = excited) they felt. We decided to use these two dimensions to ensure that participants rated their own experiences rather than the emotion expressed by the music as the selected pieces were chosen to be unambiguously happy or sad; intensity ratings of distinct emotion categories could have induced demand characteristics (i.e., participants rating the emotion expressed rather than their own experience; see Zentner & Eerola, 2010 for a detailed discussion). The experiment lasted approximately 40 min (from scheduled arrival until payout).

#### Data analysis

To prepare the video recordings for analysis with *FaceReader*, the video streams of all four cameras were first synchronized with each other using the multi-track feature in Adobe Premiere Pro CC 2018 (Version 12.1, Kentos). Start and end of the individual music pieces were identified through inspection of the sound waves. Using the zoom function in Adobe Premier, the video streams were edited to generate four separate videos (for each piece of music) of the face of each of the 50 participants (200 videos in total). Each video was exported in .mp4 format (50 frames per second, Codec H.264). Subsequently, all videos were imported into *FaceReader* (Noldus, 2016) for analysis.

This analysis in *FaceReader* followed three steps: First, for each frame of each video, the face was detected, and a 3-D model of the face created based on approximately 500 key points on the surface of the face; these are located around the eyes, eyebrows, corners of the mouth and other recognizable features. Second, from frame-to-frame, the distance between the key points was then determined. Finally, the *FaceReader* algorithm categorized facial expressions based on changes between these key points and provided a probabilistic estimate (between 0 = no expression, 1 = expression present) of the likelihood of the presence of seven different expressions (happiness, sadness, anger, surprise, fear, disgust, neutral) in each frame of the video of the face.

#### FaceReader performance

Before expressions are evaluated, *FaceReader* fits a face model to the face present in the frame. However, it is not always possible to correctly fit this model, for example if the face is temporarily obscured by the participant’s hand or other object, the participant looks away from the front of the auditorium, etc. As such, it is not always possible to derive facial expressions from every frame of the full video of each face during each piece. In order to adopt a conservative approach in our analysis of face expressions, we only included in subsequent analyses the 27 participants whose faces were detected for more than 95% of the time in each of the four pieces.

**Fig. 3:**
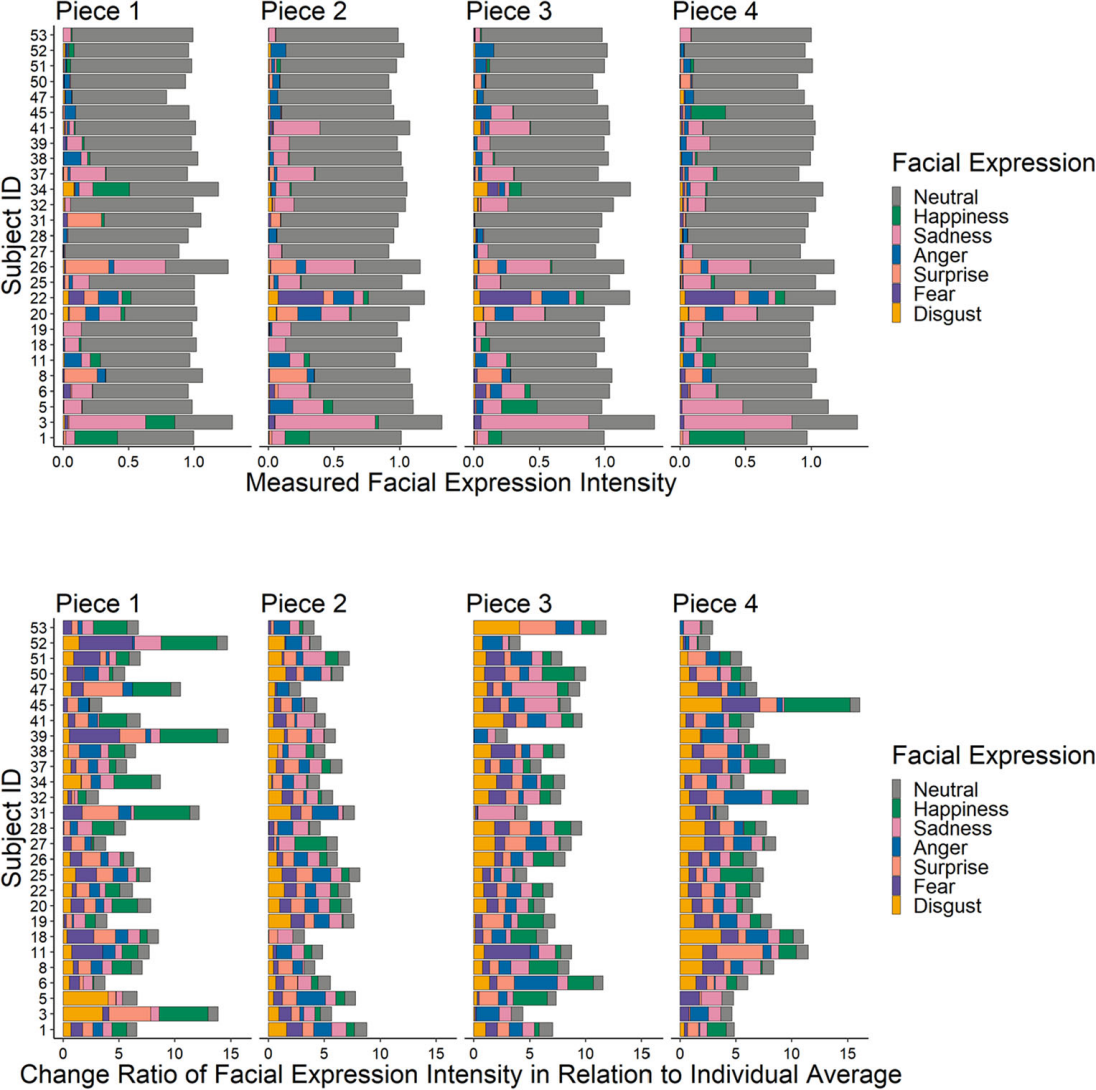
*Top*: Measured (raw) facial expression intensity values for each participant during each of the four pieces. *Bottom*: Change ratio of facial expression intensity for each piece in relation to participants’ respective facial expression averaged across all pieces

Although the term ‘neutral’ is widely used in connection to facial expressions, evidence suggests that due to their structure some faces categorized as neutral can have a subtle resemblance to emotion expression (Said et al., 2009). Hester (2018) showed that both men and women tend to show traces of negative emotion in their supposedly neutral expression, a phenomenon that is colloquially referred to as “Resting Bitch Face” (RBF, e.g., Barker, 2019; Hester, 2018). That allegedly neutral faces may also resemble positive emotion expressions was suggested by Lewinsky (Lewinski, 2015). Considering these findings, we decided to take these individual differences in facial expressions into account before data were analyzed, by normalizing expression values. First, we calculated the mean of each expression (neutral, happy, sad, anger, surprise, fear, disgust) for each piece and participant separately. Then, we calculated the overall mean of each expression within each participant for all four pieces together. Finally, for each participant, mean expressions from each piece were each divided by this overall participant mean (separately for each emotion). The resulting change ratio can be understood as deviations from the baseline, if a participant had, for example, high values for anger across all stimuli this would indicate a predisposition toward a negative neutral facial expression for that participant, thus, high values for anger in one stimulus are weighted less heavily and are considered in relation to the baseline for that expression. Figure 3 shows intensity values for facial expressions for each participant and each piece as well as the change ration for each participant and each piece.

### Results

Our first aim was to investigate whether facial expressions of multiple audience members in a concert setting can be detected. Our results show that *FaceReader* was able to fit a face model for greater than 95% of the time in 74.5% percent of all videos (summarized in Table 4).

**Table 4:** Numbers and percentages of participant faces recognized for each piece based on 95% threshold

	Piece 1	Piece 2	Piece 3	Piece 4	Total
Number of faces recognized	42	41	32	34	149
Percentage of faces recognized	84%	82%	64%	68%	74.5%
Total number of faces	50	50	50	50	200

We visually inspected *FaceReader* analyses to determine why model fit was not successful for the remaining faces and found that errors in fitting a face model or face detection occurred typically because the participant wore glasses or faces were obstructed by the participant’s hand or hair. For 27 participants the face model was fit successfully (> 95%) for all four pieces, data from these individuals are included in subsequent analyses.

**Fig. 4:**
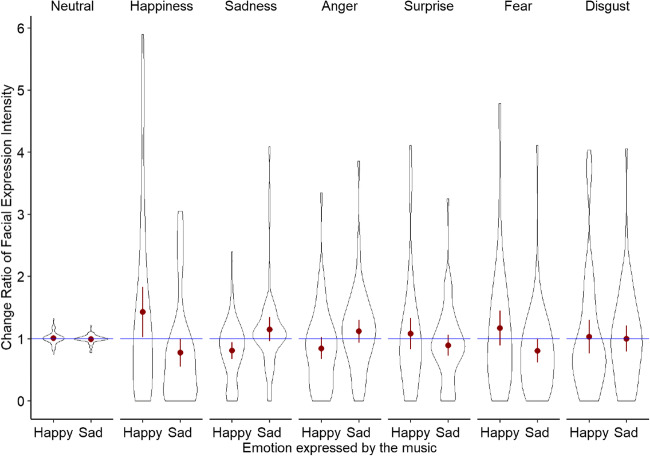
Change ratio for each facial expression by emotion expressed in music. *Red dots* indicate means, *lines* indicate 95% confidence intervals. *Values above the blue line* indicate an increase, *values below the blue line* indicate a decrease

Our second aim was to investigate whether the emotion expressed in the music predicts specific facial expressions in the audience (Fig. 4). We explored this by fitting seven separate hierarchical linear models for all facial expression categories (neutral, happy, sad, fear, surprise, anger, disgust) as dependent variables. In each model, participant identity was the upper level (“subjects” in SPSS MIXED) and time (i.e., order in which pieces were played) as the lower level (“repeated”). A new grouping variable “emotion expressed”, which codes whether a piece expressed happiness or sadness, was used as a fixed factor. The covariance structure with the best model fit (as determined by AIC) was CSH. This analysis showed that the emotion expressed in the music had a significant main effect on facial expressions of happiness and sadness. We further observed a non-significant trend for facial expressions of fear (see Table 5).

**Table 5:** Type III test for facial expressions with emotion expressed as factor

Facial Expression	Source	Numerator df	Denominator *df*	*F*	*p*
Neutral	Intercept	1	66.41	0.010	.920
	Emotion expressed	1	79.48	0.125	.724
Happy	Intercept	1	44.64	0.182	.672
	Emotion expressed	1	79.37	9.360	.003**
Sad	Intercept	1	29.647	.048	.827
	Emotion expressed	1	78.67	5.28	.024*
Anger	Intercept	1	52.073	.036	.851
	Emotion expressed	1	79.749	2.213	.141
Surprise	Intercept	1	25.538	.095	.761
	Emotion expressed	1	75.977	1.427	.236
Fear	Intercept	1	36.375	0.844	.364
	Emotion expressed	1	78.645	3.025	.086
Disgust	Intercept	1	31.793	.006	.941
	Emotion expressed	1	79.578	0.246	.621

Note. Standardized values of facial expression scores have been used for this analysis

Parameter estimates of fixed effects further show that music expressed happiness predicted an increase of facial expressions of happiness (*t*(76.86) = 2.39, *p* = .020) and a decrease of facial expressions of sadness (*t*(79.37) = – 3.06, *p* = .003). Music that expressed sadness predicted significantly greater facial expressions of sadness (*t*(78.67) = 2.30, *p* = .024) and significantly fewer facial expressions of happiness (*t*(73.25) = – 2.07, *p* = .042). In addition, we observed a non-significant trend that suggests that music that expressed sadness resulted in a decrease of facial expressions of fear (*t*(78.65) = – 1.74, *p* = .086).

**Fig. 5:**
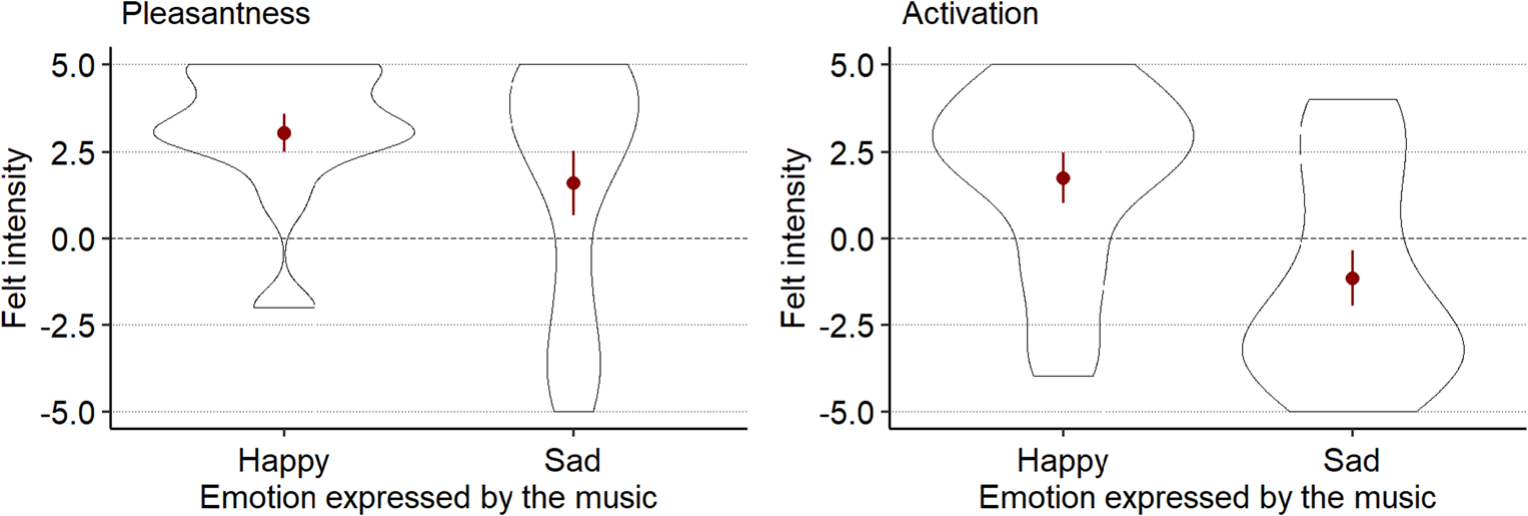
Ratings of felt intensity, by participants in the concert, of pleasantness (*left*) and activation (*right*) by emotions expressed in music. *Red dots* indicate means, *lines* indicate 95% confidence intervals

Subsequently, we tested if there were significant differences in intensity ratings for pleasantness and activation between the happy and sad pieces (Fig. 5). We fitted separate hierarchical linear models for activation and pleasantness as respective dependent variables and followed the same steps as described in the previous section, using the CSH covariance structure in both cases. Type III tests of fixed effects show that both pleasantness (*F*(1, 71.14) = 7.93, *p* = .006) as well as activation (*F*(1, 77.06) = 40.31, *p* < .001) were rated significantly higher for the happy pieces than for the sad pieces.

**Fig. 6:**
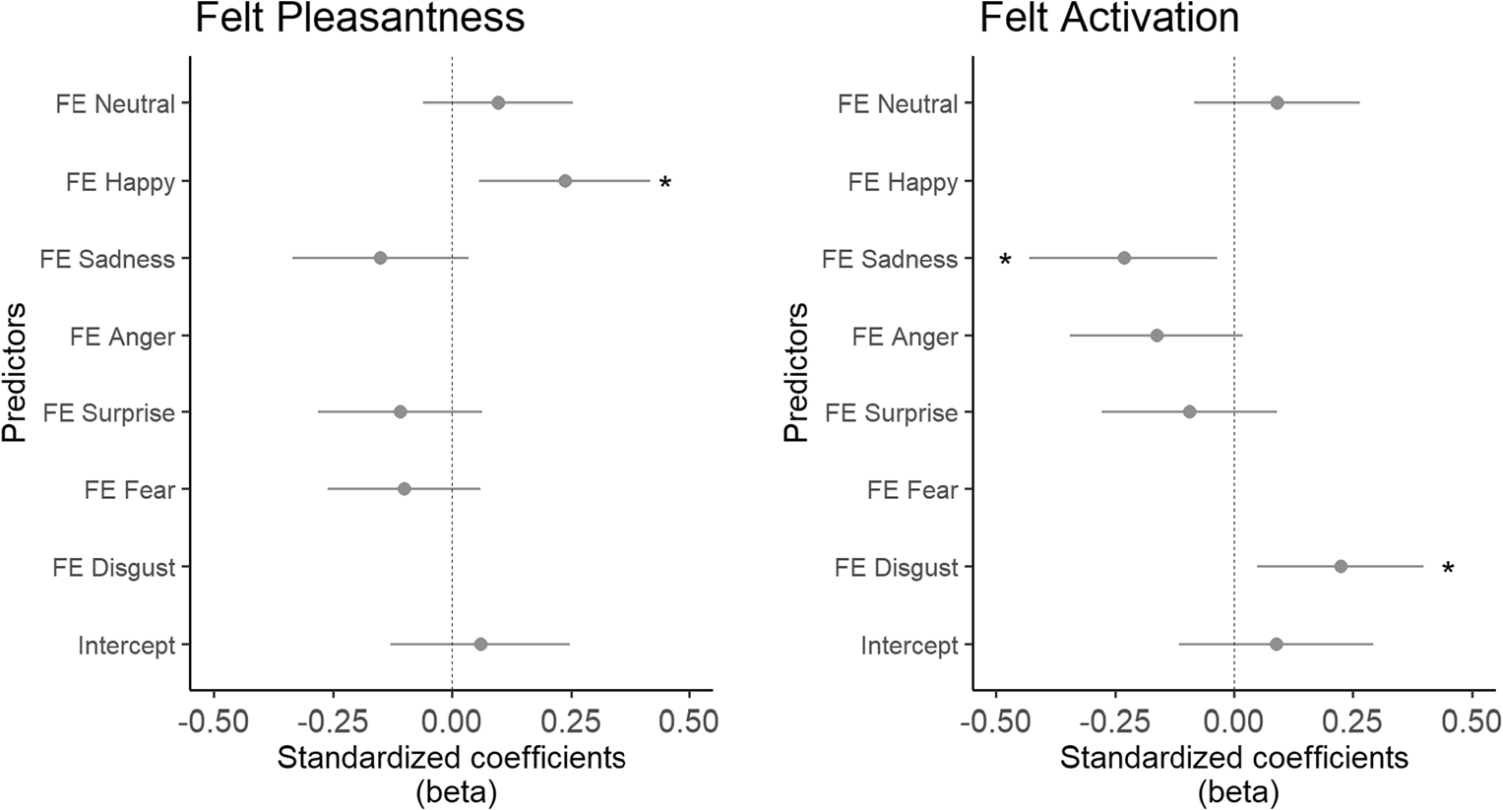
Beta-coefficients of linear mixed effects model for predicting retrospective ratings of Activation and Pleasantness. As predictors that had a *t*-value lower than 1 in the initial model were excluded from the preferred model, no beta-coefficients for these predictors were plotted. FE = Facial Expression. *p < .05

Finally, we explored whether facial expressions of emotion categorized by automated face analysis software could predict subjective ratings of pleasantness and activation. We fitted two separate hierarchical linear models for pleasantness and activation as dependent variables with participant identity as upper level and time (i.e., order in which pieces were played) as lower level. All facial expressions (neutral, happy, sad, anger, surprise, fear, disgust) were initially included as predictor variables. In a second step, predictor variables that had a *t*-value lower than 1 were excluded to improve model fit. The AIC difference between the full model and the reduced model was more than 5 for both dependent variables, which suggests that the reduced model was a better fit in both instances (see Burnham & Anderson, 2004, for an overview on model selection). Figure 6 shows the standardized beta-coefficients for predicting ratings of activation and pleasantness, respectively, *t*-values and associated *p* values for both models are summarized in Table 6.

**Table 6:** Hierarchical linear models for predicting retrospective subjective ratings of felt activation and felt pleasantness

	Pleasantness					Activation				
Predictor	*β*	95% Confidence Interval of *β*		*t*	*p*(*t*)	*β*	95% Confidence Interval of *β*		*t*	*p*(*t*)
		Lower	Upper				Lower	Upper		
Intercept	0.06	– 0.13	0.25	0.65	.524	0.09	– 0.12	0.29	0.90	.376
FE Neutral	0.10	– 0.06	0.25	1.24	.219	0.09	– 0.08	0.27	1.05	.30
FE Happy	0.24	0.06	0.42	2.63	.011*					
FE Sad	– 0.15	– 0.33	0.04	– 1.62	.109	– 0.23	– 0.43	– 0.03	– 2.35	.022*
FE Anger						– 0.16	– 0.35	0.02	– 1.79	.079
FE Surprise	– 0.11	– 0.28	0.06	– 1.26	.211	– 0.09	– 0.28	0.09	– 1.01	.315
FE Fear	– 0.10	– 0.26	0.06	– 1.27	.212					
FE Disgust						0.22	0.05	0.40	2.57	.013*

*Note. FE* = Facial Expression. Cells are left blank when predictors had a *t* lower than 1 in the initial model and were excluded from the preferred model

This analysis shows that an increase in subjectively experienced activation could be predicted by lower values for facial expressions of sadness, as well as higher values for facial expressions of disgust. Higher ratings of pleasantness were predicted by more facial expressions of happiness. Other predictor variables did not have a significant effect on either dependent variable.

### Discussion

This study is the first to explore the application of automated face analysis software for studying emotional responses to music in a concert audience. Our results show that *FaceReader* could detect faces in a group of participants and that the facial expressions identified by the software reflected the emotion expressed in the music. We further show that subjective experiences of pleasantness and activation can be predicted by distinct facial expressions of emotion. These data have the potential to contribute to the development of research methods for studying emotions in a concert audience by minimizing interference with their overall experience.

Music that expressed happiness and sadness had a significant effect on facial expressions of happiness and sadness, but not on neutral expressions or expressions of anger, fear, surprise, or disgust. Audience members displayed greater facial expressions of sadness (compared to happiness) during music that was selected to express sadness, whereas pieces expressive of happiness resulted in greater facial expressions of happiness (compared to sadness). Our findings corroborate Weth et al.’s (2015) study of participants in the laboratory. Also using *FaceReader*, they found that significantly more facial expressions of sadness were evoked by self-selected sad music as compared to the experimenter-selected happy music but found no differences in facial expressions of sadness between the sad and happy piece selected by the experimenters. However, Weth et al. (2015) report that 94% of the sad pieces selected by participants contained lyrics, but do not provide details concerning the verbal content. It is likely that the lyrics contained emotive language and might have amplified the motor response, as well as their subjective ratings. Furthermore, participants had a high preference for and familiarity with the music they wished to listen to during the experiment, other factors that have been found to influence subjectively felt intensity as well as psychophysiological activation (Grewe et al., 2009; Salimpoor et al., 2009). It is important to note that for our study only instrumental music was used, participants were screened for preferences for classical music, and in addition did not have influence on, or prior knowledge of, the music that was selected to be played at the concert. We can therefore infer that the differences we found in regard to facial expression categories are likely to be attributed to the emotions expressed in the music rather than to extramusical factors. However, we also found that facial expressions of fear were close to reaching significance. Considering the negative direction of this trend (music that expressed sadness predicted lower facial expressions of fear), this could be linked to a decrease in activation, however, fear does not appear to be a predictor for subjective ratings of activation. In addition, research by (Skiendziel et al., 2019) shows that FaceReader classifies facial expressions of fear only 51.25% of the time, whereas classification rates for all other expressions range between 75% and 100%.

Another important finding was that facial expressions of happiness predicted higher ratings of pleasantness (irrespective of the emotion expressed in the music), which is consistent with findings that associate facial expressions of happiness with positive valence (e.g. Sutton et al., 2019). Happiness is the only emotion category detected by *FaceReader* that is linked to positive valence, whereas several expressions (sadness, anger, fear, disgust) are associated with negative valence, which perhaps explains why we could not find a relationship between subjective feelings of unpleasantness and facial expressions. This accords with earlier observations, which showed that activation of the corrugator supercilii, a facial muscle that is involved in those facial expressions of emotion associated with negative valence, could predict negative valence but not be used to differentiate between distinct emotion categories (Mehu & Scherer, 2015). Thus, maybe we were unable to predict negative valenced subjective feelings as these are reflected by the activation of a muscle that is involved in a variety of facial expressions of emotion recognized by *FaceReader*. In addition, emotional processing occurs on different levels. Whereas facial mimicry is an automatic response to the stimulus, self-report is influenced by higher level cognitive processes. Matsumoto (1987) showed that facial feedback only accounted for 11.76% or less of the total variance in self-reported emotion, which indicates that individual differences could moderate the effect of automatic facial mimicry and self-reported feelings. Sonnby-Borgström (2002) found that subjects in the high-empathy group showed lower zygomaticus activity when reporting more negative feelings, whereas subjects in the low-empathy group showed higher zygomaticus activity when reporting more negative feelings. We did not measure empathy in our study, however taken together with recent findings that show that enjoyment of sad music is linked to high empathy (Vuoskoski et al., 2012), we can speculate that more facial expressions of sadness (which were associated with sad music) did not predict lower ratings of pleasantness because of individual differences such as empathy.

For ratings of activation, our results showed that as expressions of sadness increased, participants reported less activation. These findings corroborate previous studies that found an association between low levels of arousal and musical sadness (Kreutz et al., 2008), as well as minor mode and slow tempo which are musical features commonly associated with sad music (Husain et al., 2002). In our study, high ratings of activation could be predicted by more facial expressions of disgust. As high levels of arousal as well as facial expression of disgust can be attributed to an aversive response, finding this relationship is perhaps not surprising. Disgust is an emotion that is not commonly attributed to music (Vuoskoski & Eerola, 2011), however, this could be because at this point relatively little research on negative emotional experiences in music is available (e.g., Belfi & Loui, 2019; Martínez-Molina et al., 2016).

Finally, our results show that automated face analysis software could detect faces in an audience in an ecologically valid listening environment with relatively poor lighting compared to a controlled laboratory environment. The impact this approach has on valuable resources such as preparation time and manpower is apparent when comparing with Egermann et al.’s (2013) concert experiment. Here, they recorded facial EMG and other physiological signals in an audience equal in size to our sample and reported that one hour in preparation time and ten research assistants were required to connect all sensors. Therefore, participants had to wait for a substantial amount of time waiting for the experiment to begin, and along with the invasive nature of the recording equipment, would likely have an influence on their overall experience, as well as their motivation to participate in future studies. Our approach only requires a video camera which makes it possible to set up an experiment in a short amount of time and begin the experiment as soon as participants have taken their seats.

#### Limitations

Although we found that automated face analysis software detected facial expressions that reflected the emotion expressed in the music, these findings were limited to a subset of the audience. *FaceReader* is particularly sensitive to light conditions and as the venue’s light sources were mainly located in the ceiling, this resulted in shadows below the faces for some participants. In other instances, participants’ faces were partially covered by their hands or hair, issues that lead the algorithm to fail to detect the facial outline. Also faces of participants wearing glasses with big frames were not always recognized properly. The number of faces that had to be excluded increased over time, and visual inspection of video recordings showed audience behavior that could be indicative of boredom or fatigue (e.g., increases in resting head in hands and slouching). This behavior may be specific to our participants who might have experienced this concert-experiment as a chore and had a different motivation to see the concert than audience members who typically decide to attend a specific event. To ensure a greater number of detected facial expressions for any given audience size, there are a number of different actions that could be taken, although they all have implications. First, in order to retain more participants, a solution could be decrease ecological validity of the audience experience and ask participants to remove their glasses, not touch their faces or remain seated in an upright position. This would improve face detection, but probably would result in participants becoming uncomfortable over time. Second, testing audiences at live concerts, rather than more sterile environments, may limit boredom and some of these effects. Third, participant faces could be illuminated with additional auditorium lights to reduce lighting artifacts and increase face expression detection. Indeed, this approach has proved very effective in other unpublished data of ours where we measure audience facial expressions in similar auditoriums. Alternatively, an approach may be to accept the limitations of the technique and film a larger number of participants whilst retaining a more ecologically valid setting.

Automated face analysis can only detect visible movements, which may make this method less precise than fEMG which detects muscle activation directly. However, we were still able to capture very brief and subtle movements which are not easily observable by the human eye as even small muscle activation leads to detectable movement of the skin above the muscle.

Although, for the sake of simplicity and to ensure that participants remembered emotions during 4 separate pieces, we only measured subjective emotional experience using two dimensions (activation and pleasantness). However, in the future, particularly when examining the relationship between felt emotions and emotional expressions of single musical pieces, measurement of felt emotions can be considered through complex multi-dimensional models.

### Conclusions

In conclusion, this study showed that video recordings and automated face analysis software can be used to study audiences’ emotional responses to music in an ecologically valid environment. The study was conducted in a classical concert, a setting where movement is not only restricted because audience members sit down during the performance, but also because classical concert etiquette requires individuals to suppress any movement or noises so that the music can be enjoyed by others undisturbed. The present findings show that under these conditions we could detect facial expressions of emotion that reflected the emotion expressed in the music. As no special equipment is needed and only a video camera required for data collection our method also has considerable advantages when compared to invasive sensor-based methods that have been used to measure facial muscle activation in audiences. These benefits, along with the minimal time expenditure involved, could make this method particular useful for studying audience emotional responses in the future.

The datasets generated during and/or analysed during the current study are available in the [NAME] repository, https://osf.io/ztcrh/?view_only=9c31147cb1c347c59b7bfef2a5551b1c.
